# Strategies for Preventing Endoscopic Recurrence of Crohn's Disease 1 Year after Surgery: A Network Meta-Analysis

**DOI:** 10.1155/2017/7896160

**Published:** 2017-05-28

**Authors:** Jin-shan Feng, Jin-yu Li, Xiu-yan Chen, Zheng Yang, Shang-hai Li

**Affiliations:** ^1^College of Pharmacy, Guangdong Medical University, Zhanjiang 524023, China; ^2^Science Research Center (Campus Zhanjiang), Guangdong Medical University, Zhanjiang 524023, China; ^3^First School of Clinical Medicine, Guangdong Medical University, Zhanjiang 524023, China; ^4^Department of Psychology, Affiliated Hospital of Guangdong Medical University, Zhanjiang 524023, China; ^5^Department of Cardiology, Affiliated Hospital of Guangdong Medical University, Zhanjiang 524023, China

## Abstract

**Objective:**

To assess the benefits of different treatments that aim to prevent the endoscopic recurrence of Crohn's disease (CD) after ileal resection.

**Methods:**

Randomized controlled trials (RCTs) were searched from MEDLINE, Embase, and the Cochrane Central Database. All the included RCTs with an endoscopic recurrence outcome which was defined as Rutgeerts' score ≥ i2 have a duration of more than 1 year. The quality of the included RCTs was assessed by the Cochrane Risk of Bias Tool. Pairwise treatment effects were estimated through a Bayesian random effects network meta-analysis by using the OpenBUGS 1.4 software and reported as odds ratios (ORs) with a 95% credible interval (CI).

**Results:**

Fourteen RCTs (877 participants) were included. Two strategies were superior to placebo for preventing endoscopic recurrence of CD at 1 year after surgery: infliximab (d, −5.475; 95% CI, −10.47 to –1.632) and adalimumab (d, −7.273; 95% CI, −13.84 to −2.585). Nine strategies were not effective: budesnoid, mesalazine (in both high and low dose), azathioprine, *Tripterygium wilfordii,* mesalazine + infliximab, ornidazole, untreated intervention, and Lactobacillus GG.

**Conclusions:**

Except for infliximab and adalimumab, other strategies included in our analysis were not effective for preventing endoscopic recurrence of CD at 1 year after ileal resection.

## 1. Introduction

Crohn's disease (CD) is a chronic inflammatory condition of the intestinal tract that affects individuals in the prime of their lives. Over the past few decades, although the rate of intestinal resections for CD has been reduced after the introduction of immunosuppressant drugs and then antitumor necrosis factor (TNF) therapy [1], the recurrence of CD postoperation was still a challenge for the management of inflammatory bowel disease (IBD). The strategy chosen for preventing recurrent CD postoperation remains a difficult problem. Although multiple trials exist, most are placebo controlled, with a lack of head-to-head trials between active treatments. The paucity of head-to-head clinical trials has raised controversial therapeutic decisions including the choice between conventional drugs, immunosuppressants, and biological agents. According to this, comparisons of preventive strategies for CD recurrence after surgery were important for the benefit of CD patients.

In the management of postoperative CD patients, the maximized efficacy, minimized toxicity, and optimized costs should be considered; indirect evidence may help inform decision making while direct head-to-head evidence is lacking. An indirect comparison can be made between 2 treatments if each treatment has been compared with that of a common comparator. Bayesian network meta-analysis (NMA) considers all indirect and direct evidence, to determine the relative treatment effects between all interventions that can be linked through shared comparators. Considering indirect evidence adds strength to the estimation of treatment effects, even where head-to-head trials are available.

The objective of this study was to compare the efficacy of therapies for preventing CD recurrence after surgery, including mesalazine [2–8], budesonide [9, 10], azathioprine [4, 8, 11], ornidazole [12], infliximab [6, 13, 14], adalimumab [8, 14], *Tripterygium wilfordii* Hook. f. [5, 11], Lactobacillus GG [15], and untreated intervention [7], alone or combination therapies preventing CD recurrence after surgery, based on direct and indirect evidence from RCTs.

## 2. Methods

### 2.1. Eligibility Criteria

RCTs that assessed treatments in postoperative patients with CD were included, and trials assessing endoscopic recurrence had to be at least 48 weeks in duration [16]. The primary outcome was endoscopic recurrence, which was defined as Rutgeerts' score ≥ i2. When trials did not report Rutgeerts' score, trials studying nonpostoperative patients, trials in which the treatment was not fixed (e.g., standard of care), trials with a randomized withdrawal design, trials with a crossover design, trials exclusively assessing fistulizing CD, and trials that did not report recurrence as an outcome were excluded.

### 2.2. Literature Search and Study Selection

The RCTs were searched in MEDLINE, Embase, and the Cochrane Central Register of Controlled Trials. The database search strategy was adapted from a systematic reviews [17] (the full search strategy is shown in Supplementary Table 4 available online at https://doi.org/10.1155/2017/7896160). Meanwhile, we also have a search of trial registries (www.clinicaltrials.gov) and by screening all the American College of Gastroenterology, Digestive Disease Week, United European Gastroenterology Week, and European Crohn's and Colitis Organization conference. Search results were screened by 2 independent reviewers (X. C., J. L.) first by title and abstract and then by full text. Disagreements were resolved through consensus and discussion with a third reviewer (J. F.). Selected studies were reviewed by 2 reviewers (Z. Y., S. L.).

### 2.3. Data Collection and Quality Appraisal

Data were abstracted for relevant study characteristics (Supplementary Table 1) and for the outcome of endoscopic recurrence. The number of endoscopic recurrences was extracted at the end of the trial. Data were extracted on a basis of per-protocol analysis.

In each trial arm, we abstracted the total number of patients randomized and the total number of patients who experienced the outcome. If only percentages were reported, the number of patients with the outcome was calculated and rounded to the nearest whole number. Any disagreements were resolved through discussion and repeat extraction. The quality of trials was rated through the Cochrane Risk of Bias tool.

### 2.4. Synthesis of Results

NMA was conducted to compare multiple interventions simultaneously for the outcome of endoscopic recurrence of CD postoperation. NMA combines direct evidence within trials and indirect evidence across trials [18]. A network plot obtained to ensure that the trials were connected by treatments using Stata 13 software (StataCorp LP). We excluded any RCTs that were not connected to the network. A Bayesian NMA was conducted using the Markov chain Monte Carlo method in OpenBUGS 1.4 software. Both random effects models [19] and fixed effects models [20] were used for NMA, and the model with lower DIC is generally chosen to aid better interpretation as it takes the model complexity into account [21]. In our analysis, the difference in DIC between the random effects model and the fixed effects model was less than 3 (Supplementary Table 2); considering that the heterogeneity of the RCTs included in this analysis was more than 50%, the random effects model was chosen for recurrent prevention. The code of Bayesian NMA used in the OpenBUGS software has been published [22] and is provided in the Supplementary Materials. Statistical heterogeneity analyses were performed using RevMan 5.3 software.

Uninformative prior probability distributions were used for variables. All chains were run with 10,000 burn-in iterations followed by 40,000 monitoring iterations. Convergence was assessed by running 3 chains.

The probability that each intervention ranks at one of the possible positions was estimated. The probability that a treatment ranks as the best treatment was presented. It should be noted that a less than 90% probability that the treatment is the best treatment is unreliable [23]. The cumulative probability of the treatment ranks (rankogram) was also presented.

### 2.5. Summary Measures

For each pairwise comparison, the OR were calculated with a 95% credible interval (CI) and the probability that each treatment was superior to the other. We considered a treatment as showing superiority (or inferiority) if the 2-sided 95% CI of the OR excluded 0, which equates to a 97.5% probability that the treatment is superior.

## 3. Results

### 3.1. Search Results, Trial Characteristics, and Risk of Bias

**Figure 1:**
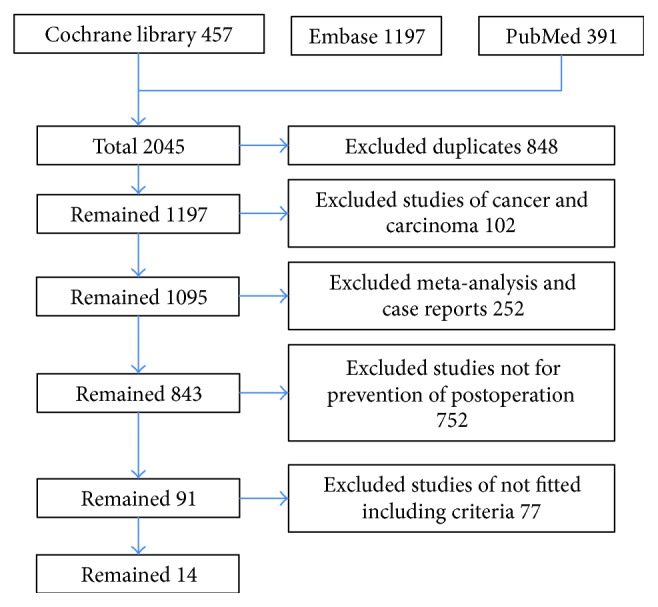
Study flow diagram.

**Figure 2:**
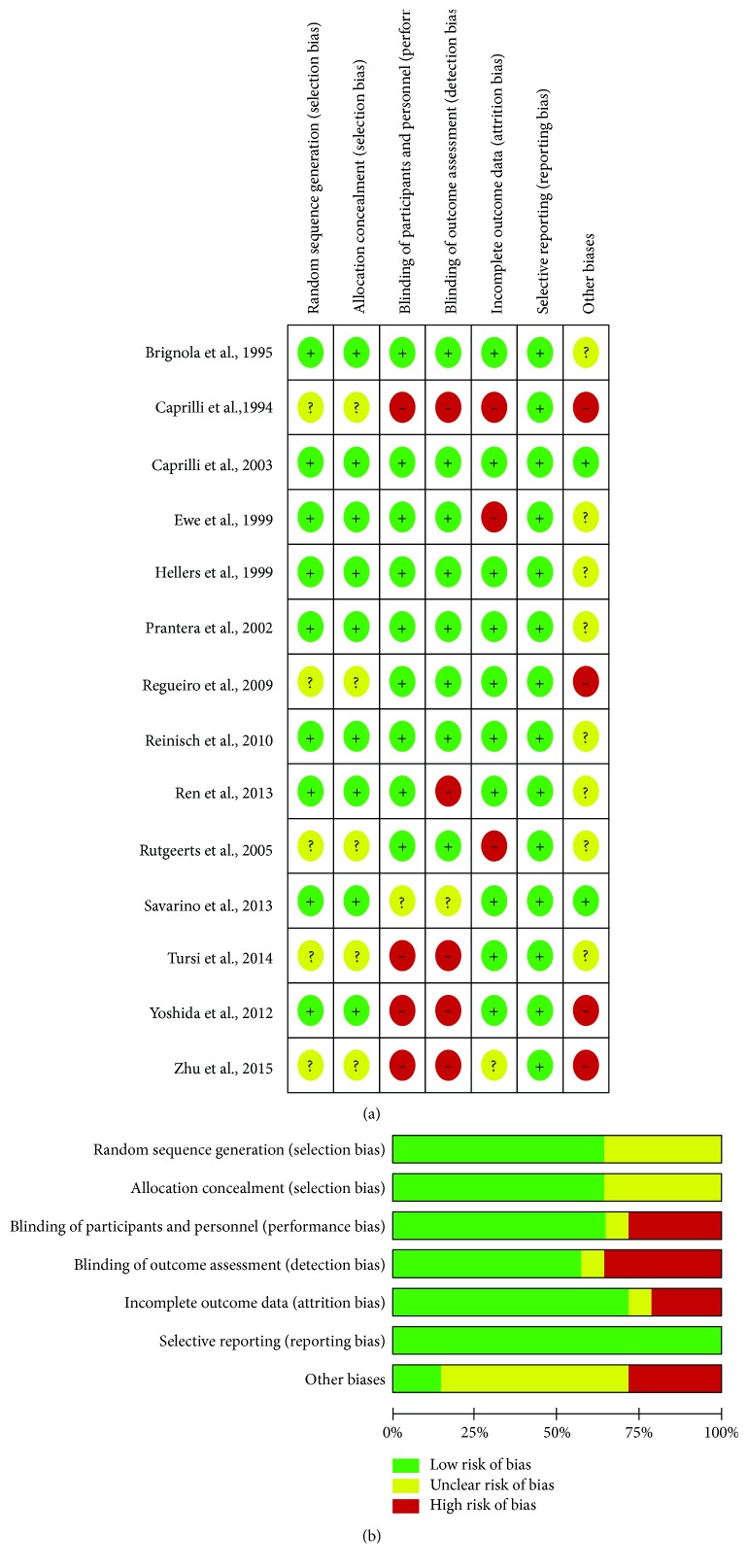
The risk of bias of the included trials. (a) Risk of bias summary: each risk of bias item for each included study; (b) risk of bias graph: each risk of bias item is presented as percentages across all included studies.

Fourteen trials (877 participants) were included (Supplementary Table 1). Two trials evaluated the traditional Chinese medicine and glucocorticoid therapy; 4 trials evaluated anti-TNF therapy, 3 trials evaluated immunosuppressants, 7 trials evaluated 5-aminosalicylic acid therapy, and 1 trial evaluated untreated intervention, nitroimidazole agent, and probiotic agent therapy. Seven trials compared active treatments and did not include a placebo or untreated intervention arm. The study flow diagram is shown in Figure 1. The risk of bias of the included trials is shown in Figure 2. The characteristics of included trials were shown in Supplementary Table 1. Six trials were not mentioned in the anastomosis of the surgery; 5 trials were the side-to-side and stapled anastomosis; 1 trial was the end-to-end and hand-sewn anastomosis; 2 trials were the multianastomosis.

### 3.2. Synthesis of Results

**Figure 3:**
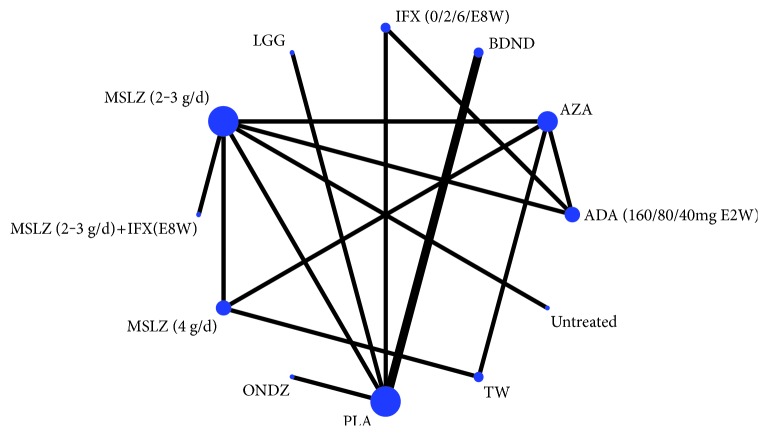
Network diagram. The size of the nodes was according to the number of patients that have received each treatment and the edges according to the mean control group risk for all comparisons versus placebo. PLA, placebo; untreated, blank control group; MSLZ, mesalazine; BDND, budesonide; AZA, azathioprine; IFX, infliximab; ADA, adalimumab; TW, *Tripterygium wilfordii*; LGG, Lactobacillus GG.

**Figure 4:**
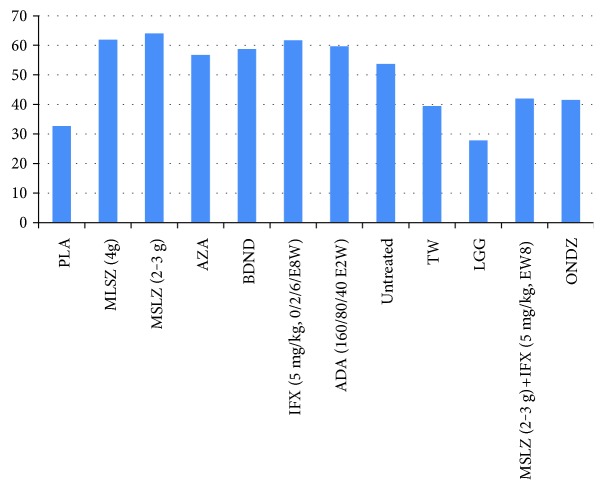
Rankogram. This diagram shows the probability that the treatment is best for CD recurrence. None of the treatments appear clearly superior to others when all the outcomes are considered together. There does not seem to be much correlation between a treatment being best in reducing blood transfusion and a treatment being best in reducing recurrent events. PLA, placebo; untreated, blank control group; MSLZ, mesalazine; BDND, budesonide; AZA, azathioprine; IFX, infliximab; ADA, adalimumab; TW, *Tripterygium wilfordii*; LGG, Lactobacillus GG.

Two strategies were superior to placebo for preventing endoscopic recurrence of CD at 1 year after surgery: infliximab (0/2/6/E8W) (d, −5.475; 95% CI, −10.47 to −1.632) and adalimumab (160/80/40 mg, E2W) (d, −7.273; 95% CI, −13.84 to −2.585). Nine strategies were not effective: budesnoid (d, −0.4136; 95% CI, −3.051 to 2.152); mesalazine 4 g/d (d, −0.845; 95% CI, −4.705 to 4.443); mesalazine 2-3 g/d (d, −1.053; 95% CI, −4.16 to 3.03); azathioprine (d, −1.389; 95% CI, −5.236 to 3.899); *Tripterygium wilfordii* (d, −1.174; 95% CI, −5.73 to 4.581); mesalazine (2-3 g/d) + infliximab (E8W) (d, −3.795; 95% CI, −9.191 to 3.016); ornidazole (d, −1.818; 95% CI, −5.519 to 1.87); untreated intervention (d, −0.058; 95% CI, −4.637 to 5.522); and Lactobacillus GG (d, 1.062, 95% CI, −2.81 to 4.86) (Table 1; Supplementary Table 2 and Figure 3()). The most effective strategy was not found (Figure 4(), Supplementary Table 3).

## 4. Discussion

Many patients with CD require surgical resection of their disease at some stage. After surgery, the disease tends to occur in a similar fashion and usually within a short time. If left untreated, approximately 80% of patients will have an endoscopic recurrence within 1 year from surgery; and in a large majority of them, the disease will manifest clinically within a variable period of time [17]. It is clear that postoperative recurrence is a major problem in the management of patients with CD. A range of interventions has been suggested to reduce postoperative recurrence, and many have been evaluated by RCTs. This review critically evaluates the published data in this area.

By applying a NMA approach, our study integrated direct head-to-head data with indirect evidence to provide the most robust data available for preventing the recurrence of CD after surgery. Until more head-to-head trials are completed, these data are the best we have available to guide clinical decision making within and between classes of drug therapies. A concern with any meta-analysis is heterogeneity across trials. The trials in our study differed in several aspects, including the disease severity of the patient populations, risk of bias, disease severity at the time of randomization, different interventions, prior exposure to drug therapy, and primary end points. We also found consistency between the treatment effects in the NMA and those observed in a direct (traditional) meta-analysis (the forest plot is available in Supplementary Figure 1), when direct evidence was available, although there were a few closed loops in the evidence network (Figure 3). When all the strategies were compared together via rankogram, the most effective strategy could not be found (Figure 4).

The efficacy of 5-ASA drugs for the treatment of Crohn's disease has received much recent adverse scrutiny, and previous systematic reviews have not demonstrated a significant benefit for 5-ASA for the maintenance of medically induced remission in Crohn's disease. Our NMA results found that, regardless of the dose, the effect of mesalazine was not superior to placebo, which is similar to monotherapy or combined with infliximab.

Immunosuppressive medications such as azathioprine present the more difficult propositions as their toxicity are greater, an issue which is all the more important in the context of prevention rather than treatment of the disease. Another earlier meta-analysis of the effect of azathioprine or 6-mercaptopurine on postoperative recurrence also suggests that purine antimetabolites are effective [24] but were performed on the assumption that mesalazine had effects equivalent to placebo and thus failed to evaluate the comparative effects of immunosuppressive medications and 5-ASA drugs. In our analysis, we had difficulty in detecting any superiority of this agent to placebo for prevention of endoscopic recurrence. In a previous study, azathioprine or 6-mercaptopurine appeared generally effective for maintenance of remission in CD [25].

There has been much interest in the role of enteric microflora in the pathobiology of IBD, and postoperative recurrence may be influenced by the presence or absence of certain bacterial species [26]. Both probiotic species and antibiotic agents have been evaluated in the postoperative setting. While there is significant heterogeneity in the probiotic formulations which have been trialed, these studies have neither individually nor collectively demonstrated any impact on endoscopic recurrence rates. In contrast, antibiotic therapy with nitroimidazole agent such as ornidazole appeared better than placebo in traditional meta-analysis (Supplementary Figure 1) to prevent endoscopic recurrence at one year, but the results of NMA do not support that finding. Considering the issue of tolerability (predominantly due to gastrointestinal or neurological side effects), the nitroimidazole agent can be justified as an initial short-term intervention. On the other hand, the probiotic species have failed to demonstrate efficacy in a small number of trials.

The introduction of anti-TNF therapy has had a significant impact on the management of CD by improving quality of life and reducing hospitalization and surgery. A current NMA found that adalimumab and infliximab+azathioprine are the most effective therapies for induction and maintenance of remission of moderate-to-severe CD [27]. For those patients who experienced a loss of response, dose intensification has been suggested [28]. Our recent study also found that adalimumab is effective and safe in inducing remission for moderate-to-severe ulcerative colitis [24], and it had been reported that adalimumab was effective for patients with ulcerative colitis who have lost response or are intolerant of infliximab [29, 30]. In this NMA results, infliximab and adalimumab are both superior to placebo, but the efficacy of adalimumab was not superior to infliximab.

The generalizability of our results is limited to the eligible population enrolled in the included trials. For example, RCTs typically recruit CD patients who have underwent surgery. Furthermore, the surgical treatment approaches for CD may differ based on age, disease location, disease behavior, prior medication, and smoking, which could not be explored using the data available in the clinical trials [31]. Similarly, we were not able to evaluate RCTs that compared surgical strategies of treating CD such as comparing the “side-to-side” or “side-to-end” anastomosis. Meanwhile, the limitations of this review are obvious, such as whether the withdrawal rates or rates of adverse events were not assessed.

By integrating direct and indirect evidence, this NMA serves as a guide for clinicians making complicated decisions on the medical management of postoperative recurrent CD. IFX and ADA were the effective strategies at preventing endoscopic recurrence when compared with placebo and other treatments. These data call for randomized controlled head-to-head trials between commonly prescribed medication regimens for postoperative recurrent CD.

## Supplementary Material

Table 1. Characteristics of Included Trials. Table 2. Recurrence results and model fit. Table 3. Intervention measures. Figure 1. Forest plot. PLA, placebo; untreated, blank control group; MSLZ, mesalazine; BDND, budesonide; AZA, azathioprine; IFX, infliximab; ADA, adalimumab; TW, tripterygium wilfordii; LGG, lactobacillus GG. Table 4. Search strategy. Appendix: Statistical code.

## Figures and Tables

**Table 1:** Result of network meta-analysis.

	Mean	SD	MC_error	val2.5pc	Median	val97.5pc	Intervention
d[2]	−0.8045	2.202	0.0506	−4.705	−1.068	4.443	MSLZ 4 g/d
d[3]	−1.053	1.715	0.03574	−4.16	−1.201	3.03	MSLZ 2-3/d
d[4]	−1.389	2.222	0.0498	−5.236	−1.628	3.899	AZA
d[5]	−0.4136	1.244	0.007319	−3.051	−0.406	2.152	BDND
d[6]	−5.475	2.222	0.04268	−10.47	−5.287	−1.632	IFX (0/2/6/E8W)
d[7]	−7.273	2.849	0.06321	−13.84	−6.948	−2.585	ADA 160/80/40
d[8]	−0.05878	2.438	0.03836	−4.637	−0.2134	5.522	Untreated
d[9]	−1.174	2.47	0.05239	−5.73	−1.367	4.581	TW
d[10]	1.062	1.848	0.01396	−2.81	1.075	4.86	LGG
d[11]	−3.795	2.943	0.05731	−9.191	−4.021	3.016	MSLZ (2-3 g) + IFX (5 mg/kg, E8W)
d[12]	−1.818	1.757	0.01178	−5.519	−1.814	1.87	ONDZ

Untreated, blank control group; MSLZ: mesalazine; BDND: budesonide; AZA: azathioprine; IFX: infliximab; ADA: adalimumab; TW: *Tripterygium wilfordii*; LGG: Lactobacillus GG; d[2] indicates the log odds ratio between treatment 2 and treatment 1 (d[1]); d[3] indicates the log odds ratio between treatment 3 and treatment 1 and so on.

## References

[1] Frolkis A. D., Dykeman J., Negrón M. E. (2013). Risk of surgery for inflammatory bowel diseases has decreased over time: a systematic review and meta-analysis of population-based studies. *Gastroenterology*.

[2] Brignola C., Cottone M., Pera A. (1995). Mesalamine in the prevention of endoscopic recurrence after intestinal resection for Crohn’s disease. *Gastroenterology*.

[3] Caprilli R., Cottone M., Tonelli F. (2003). Two mesalazine regimens in the prevention of the post-operative recurrence of Crohn’s disease: a pragmatic, double-blind, randomized controlled trial. *Alimentary Pharmacology & Therapeutics*.

[4] Caprilli R., Cottone M., Tonelli F. (2010). Azathioprine versus mesalazine for prevention of postoperative clinical recurrence in patients with Crohn’s disease with endoscopic recurrence: efficacy and safety results of a randomised, double-blind, double-dummy, multicentre trial. *Gut*.

[5] Ren J., Wu X., Liao N. (2013). Prevention of postoperative recurrence of Crohn’s disease: Tripterygium wilfordii polyglycoside versus mesalazine. *The Journal of International Medical Research*.

[6] Yoshida K., Fukunaga K., Ikeuchi H. (2012). Scheduled infliximab monotherapy to prevent recurrence of Crohn’s disease following ileocolic or ileal resection: a 3-year prospective randomized open trial. *Inflammatory Bowel Diseases*.

[7] Caprilli R., Andreoli A., Capurso L. T. (1994). Oral mesalazine (5-aminosalicylic acid; Asacol) for the prevention of post-operative recurrence of Crohn’s disease. Gruppo Italiano per lo Studio del Colon e del Retto (GISC). *Alimentary Pharmacology & Therapeutics*.

[8] Savarino E., Bodini G., Dulbecco P. (2013). Adalimumab is more effective than azathioprine and mesalamine at preventing postoperative recurrence of Crohn’s disease: a randomized controlled trial. *American Journal of Gastroenterology*.

[9] Hellers G., Cortot A., Jewell D., The IOIBD Budesonide Study Group (1999). Oral budesonide for prevention of postsurgical recurrence in Crohn’s disease. *Gastroenterology*.

[10] Ewe K., Böttger T., Buhr H. J., Ecker K. W., Otto H. F. (1999). Low-dose budesonide treatment for prevention of postoperative recurrence of Crohn’s disease: a multicentre randomized placebo-controlled trial. German Budesonide Study Group. *European Journal of Gastroenterology & Hepatology*.

[11] Zhu W., Li Y., Gong J. (2015). Tripterygium wilfordii Hook. f. versus azathioprine for prevention of postoperative recurrence in patients with Crohn’s disease: a randomized clinical trial. *Digestive and Liver Disease: Official Journal of the Italian Society of Gastroenterology and the Italian Association for the Study of the Liver*.

[12] Rutgeerts P., Van Assche G., Vermeire S. (2005). Ornidazole for prophylaxis of postoperative Crohn’s disease recurrence: a randomized, double-blind, placebo-controlled trial. *Gastroenterology*.

[13] Regueiro M., Schraut W., Baidoo L. (2009). Infliximab prevents Crohn’s disease recurrence after ileal resection. *Gastroenterology*.

[14] Tursi A., Elisei W., Picchio M. (2014). Comparison of the effectiveness of infliximab and adalimumab in preventing postoperative recurrence in patients with Crohn’s disease: an open-label, pilot study. *Techniques in Coloproctology*.

[15] Prantera C., Scribano M. L., Falasco G., Andreoli A., Luzi C. (2002). Ineffectiveness of probiotics in preventing recurrence after curative resection for Crohn’s disease: a randomised controlled trial with Lactobacillus GG. *Gut*.

[16] Mahadevan U., Kane S. (2006). American gastroenterological association institute technical review on the use of gastrointestinal medications in pregnancy. *Gastroenterology*.

[17] Doherty G., Bennett G., Patil S., Cheifetz A., Moss A. C. (2009). Interventions for prevention of post-operative recurrence of Crohn’s disease. *Cochrane Database of Systematic Reviews*.

[18] Mills E. J., Ioannidis J. P., Thorlund K., Schunemann H. J., Puhan M. A., Guyatt G. H. (2012). How to use an article reporting a multiple treatment comparison meta-analysis. *Journal of the American Medical Association*.

[19] DerSimonian R., Laird N. (1986). Meta-analysis in clinical trials. *Controlled Clinical Trials*.

[20] Demets D. L. (1987). Methods for combining randomized clinical trials: strengths and limitations. *Statistics in Medicine.*.

[21] Dias S., Welton N. J., Sutton A. J., Ades A. (2013). *NICE DSU Technical Support Document 2: A Generalised Linear Modelling Framework for Pairwise and Network Meta-Analysis of Randomised Controlled Trials*.

[22] Ades A. E., Sculpher M., Sutton A., Abrams K., Cooper N. (2006). Bayesian methods for evidence synthesis in cost-effectiveness analysis. *PharmacoEconomics*.

[23] Dias S. (2012). *NICE DSU Technical Support Document 1: Introduction to Evidence Synthesis for Decision Making*.

[24] Yang Z., Ye X. Q., Zhu Y. Z. (2015). Short-term effect and adverse events of adalimumab versus placebo in inducing remission for moderate-to-severe ulcerative colitis: a meta-analysis. *International Journal of Clinical and Experimental Medicine*.

[25] Chande N., Patton P. H., Tsoulis D. J., Thomas B. S., MacDonald J. K. (2015). Azathioprine or 6-mercaptopurine for maintenance of remission in Crohn’s disease. *Cochrane Database of Systematic Reviews*.

[26] Sokol H., Pigneur B., Watterlot L. (2008). Faecalibacterium prausnitzii is an anti-inflammatory commensal bacterium identified by gut microbiota analysis of Crohn disease patients. *Proceedings of the National Academy of Sciences of the United States of America*.

[27] Hazlewood G. S., Rezaie A., Borman M. (2015). Comparative effectiveness of immunosuppressants and biologics for inducing and maintaining remission in Crohn’s disease: a network meta-analysis. *Gastroenterology*.

[28] Billioud V., Sandborn W. J., Peyrin-Biroulet L. (2011). Loss of response and need for adalimumab dose intensification in Crohn’s disease: a systematic review. *The American Journal of Gastroenterology*.

[29] Peyrin-Biroulet L., Laclotte C., Roblin X., Bigard M.-A. (2007). Adalimumab induction therapy for ulcerative colitis with intolerance or lost response to infliximab: an open-label study. *World Journal of Gastroenterology*.

[30] Singh A., Mahadevan U., Yen E., Terdiman J. (2009). Adalimumab for patients with ulcerative colitis who have lost response or are intolerant of infliximab: initial response rates are high, but the response may not be durable. *American Journal of Gastroenterology*.

[31] Moran G. W., Dubeau M. F., Kaplan G. G. (2014). Phenotypic features of Crohn’s disease associated with failure of medical treatment. *Clinical Gastroenterology & Hepatology the Official Clinical Practice Journal of the American Gastroenterological Association*.

