# Supplementary material for: Strategies for Preventing Endoscopic Recurrence of Crohn's Disease 1 Year after Surgery: A Network Meta-Analysis
Source: Gastroenterol Res Pract. 2017 May 28;2017:7896160. doi: 10.1155/2017/7896160 (PMC5467338; doi:10.1155/2017/7896160)
Supplement: Supplementary file 2 [file 7896160.f2.pdf]

Strategies for Preventing Endoscopic Recurrence of Crohn's Disease 1 year after Surgery: A Network Meta-analysis

Supplementary Materials

Jin-shan Feng, Jin-yu Li, Xiu-yan Chen, Zheng Yang, Shang-hai Li

1. Results

Table 1. Characteristics of Included Trials

ID	Study	Study arms	Number of randomized patients	Location; number of centers	Time of intervention start	Time of the follow up visits	Disease severity/duration at the time of randomization	Anastomosis
1	Hellers, G. (1999) [1]	PLA vs BDND (6 mg/day)	88	Belgium; 13	within 2 weeks after surgery	at surgery and weeks 13, 52 after surgery	not mentioned	Not mentioned
2	Ewe, K. (1999) [2]	PLA vs BDND (6 mg/day)	62	Germany	within 2 weeks after surgery	weeks 12, 48 after surgery	Disease duration (month) PLA group, 81±58; BDND, 100±74	Not mentioned
3	Brignola, C. (1995) [3]	PLA vs MSLZ (3 g/day)	60	Italy; 8	within 1 month after surgery	week 48	Disease duration (month) PLA group, 69±54; MSLZ, 75±73	Not mentioned
4	Regueiro, M. (2009) [4]	PLA vs IFX (5 mg/kg, 0/2/6W, E8W)	24	Pennsylvania; 1	2-4 weeks post-operation	2-4 weeks after the final 54-week study infusion	CDAI (median): PLA group, 202; IFX group,112	Side to side and stapled
5	Prantera, C. (2002) [5]	PLA vs LGG (6 billion colony forming units, bid)	32	Italy; 1	as soon as patients could take solid food by mouth 10 days after surgery	weeks 13, 26, 39, 52	Mean disease duration (years): PLA group 7.4; LGG group 6.5	Not mentioned

ID	Study	Study arms	Number of randomized patients	Location; number of centers	Time of intervention start	Time of the follow up visits	Disease severity/duration at the time of randomization	Anastomosis
6	Rutgeerts, P. (2005) [6]	PLA vs ONDZ (1 g/day)	71	Belgium; 2	within 2 wks after surgery	weeks 12, 48	duration of disease until resection (median): PLA group,3; ONDZ group, 7	End to end and hand-sewn
7	Caprilli, R. (2003) [7]	MSLZ (4 g/day) vs MSLZ (2.4 g/day)	165	Italy; 17	2 weeks after surgery	weeks 48	Mean CDAI: MSLZ 4 group, 285; MSLZ 2.4 group, 290	End to end, 42 vs 47<br>End to side, 14 vs 13<br>Side to end, 3 vs 4<br>Side to side 33 vs 36
8	Reinisch, W. (2010) [8]	MSLZ (4 g/d) vs AZA (2-2.5mg/kg/day)	58	Austria, Czech, Germany and Isreal; 21	not mantioned	1-2 week before baseline, baseline (day 0), weeks 2, 4, 8, 24, 36, 52	Screening Rutgeerts score(mean±SD) MSLZ group, 2.97±0.93; AZA group, 3.17±0.89	Not mentioned
9	Ren, J. (2013) [9]	MSLZ (4 g/day) vs TW (1 mg/kg/day)	36	China; 1	not mantioned	weeks 26, 52	"disease severity were similar"	Not mentioned
10	Yoshida, K. (2012) [10]	MSLZ (2.25-3 g/day) vs MSLZ (2.25-3 g/day)+IFX (5 mg/kg, E8W)	30	Japan; 1	within 4 weeks post-operation	weeks 48	Mean CDAI: MSLZ+IFX group, 213.1±57.5; MSLZ group,232.8±94.4	Side to side and stapled
11	Caprilli, R. (1994) [11]	MSLZ (2.4 g/day) vs untreated	95	Italy; 15	2 weeks post-operation	weeks 24, 48	Mean CDAI: MSLZ group, 326; untreated group,321	Termino-terminal, 26 vs 26<br>Termino-lateral, 9 vs 9<br>Latero-terminal, 1 vs 1<br>Latero-lateral, 11 vs 12
12	Savarino, E (2013) [12]	MSLZ (3 g/day) vs AZA (2 mg/kg/day) vs ADA (160/	51	Italy; 1	2-4 weeks post-operation	weeks 48	Mean CDAI: MSLZ group,266; AZA group, 248; ADA	Side to side and stapled

ID	Study	Study arms	Number of randomized patients	Location; number of centers	Time of intervention start	Time of the follow up visits	Disease severity/duration at the time of randomization	Anastomosis
		80 mg at 0, 2 weeks follow by 40 mg every 2 weeks)					group,268	
13	Zhu, W. (2015) [13]	AZA (2 mg/kg/day) vs TW (1.5 mg/kg/day)	85	China; 1	within 2 weeks post-operation	weeks 26, 52	Mean CDAI: AZA group, 193.15; TW group,198.95	Side to side and stapled
14	Tursi,A. (2014) [14]	IFX (5 mg/kg, 0/2/6W, E8W) vs ADA (160/ 80 mg at 0, 2 weeks follow by 40 mg every 2 weeks)	20	Italy; 1	4-6 weeks post-operation	weeks 48	not mentioned	Side to side and stapled

PLA, placebo; untreated, blank control group; MSLZ, mesalazine; BDND, budesonide; AZA, azathioprine; IFX, infliximab; ADA, adalimumab; TW, tripterygium wilfordii; LGG, lactobacillus GG

Table 2. Recurrence results and model fit

	Fixed effect model	Random effect model	
d[2]	-1.548 (95% CI -2.811 to -0.2711)	-0.8045 (95% CI -4.705 to 4.443)	MSLZ 4g/d
d[3]	-1.374 (95% CI -2.516 to -0.2526)	-1.053 (95% CI -4.16 to 3.03)	MSLZ 2-3/d
d[4]	-2.077 (95% CI -3.488 to -0.6399)	-1.389 (95% CI -5.236 to 3.899)	AZA
d[5]	-0.3742 (95% CI -1.042 to 0.2898)	-0.4136 (95% CI -3.051 to 2.152)	BDND
d[6]	-5.212 (95% CI -8.636 to -2.669)	-5.475 (95% CI -10.47 to -1.632)	IFX (0/2/6/E8W)
d[7]	-6.811 (95% CI -11.15 to -3.767)	-7.273 (95% CI -13.84 to -2.585)	ADA 160/80/40
d[8]	-0.3689 (95% CI -1.832 to 1.095)	-0.05878 (95% CI -4.637 to 5.522)	untreated
d[9]	-1.575 (95% CI -3.076 to -0.03717)	-1.174 (95% CI -5.73 to 4.581)	TW
d[10]	1.077 (95% CI -0.3786 to 2.593)	1.062 (95% CI -2.81 to 4.86)	LGG
d[11]	-4.572 (95% CI -6.981 to -2.36)	-3.795 (95% CI -9.191 to 3.016)	MSLZ(2-3g)+IFX(5mg/kg,E8W)
d[12]	-1.801 (95% CI -2.915 to -0.7502)	-1.818 (95% CI -5.519 to 1.87)	ONDZ
Dbar	133	127.8	
pD	25.01	28.01	
DIC	158	155.8	

d[2] indicates the log odds ratio between treatment 2 and treatment 1; d[3] indicates the log odds ratio between treatment 3 and treatment 1; and so on.

Dbar indicates the posterior mean of the residual deviance.

pD indicates the effective number of parameters (leverage).

DIC indicates the 'Deviance Information Criterion'.

A lower Dbar indicates a better model fit. However, a model with lower DIC is generally chosen to aid better interpretation as it takes the model complexity into account. A lower DIC indicates a better model fit. Differences of less than 3 to 5 between the models are not considered important.

Based on the above information, fixed-effect model is the preferred model. There is no evidence of inconsistency.

CI, confidence intervals; PLA, placebo; untreated, blank control group; MSLZ, mesalazine; BDND, budesonide; AZA, azathioprine; IFX, infliximab; ADA, adalimumab; TW, tripterygium wilfordii; LGG, lactobacillus GG.

Table 3. Intervention measures

Treatment	SUCRA	PrBest	MeanRank
PLA	32.7	5.3	8.4
MSLZ(4g)	61.9	30.6	5.2
MSLZ(2-3g)	64.0	26.4	5.0
AZA	56.8	12.4	5.7
BDND	58.7	7.3	5.5
IFX(5mg/kg,0/2/6/E8W)	61.7	6.0	5.2
ADA(160/80/40E2W)	59.7	5.8	5.4
UNTREATED	53.7	4.1	6.1
TW	39.4	1.7	7.7
LGG	27.8	0.3	8.9
MSLZ(2-3g)+IFX(5mg/kg,E8W)	42.0	0.0	7.4
ONDZ	41.5	0.0	7.4

None of the treatments appear clearly superior to others when all the outcomes are considered together.

PLA, placebo; untreated, blank control group; MSLZ, mesalazine; BDND, budesonide; AZA, azathioprine; IFX, infliximab; ADA, adalimumab; TW, tripterygium wilfordii; LGG, lactobacillus GG.

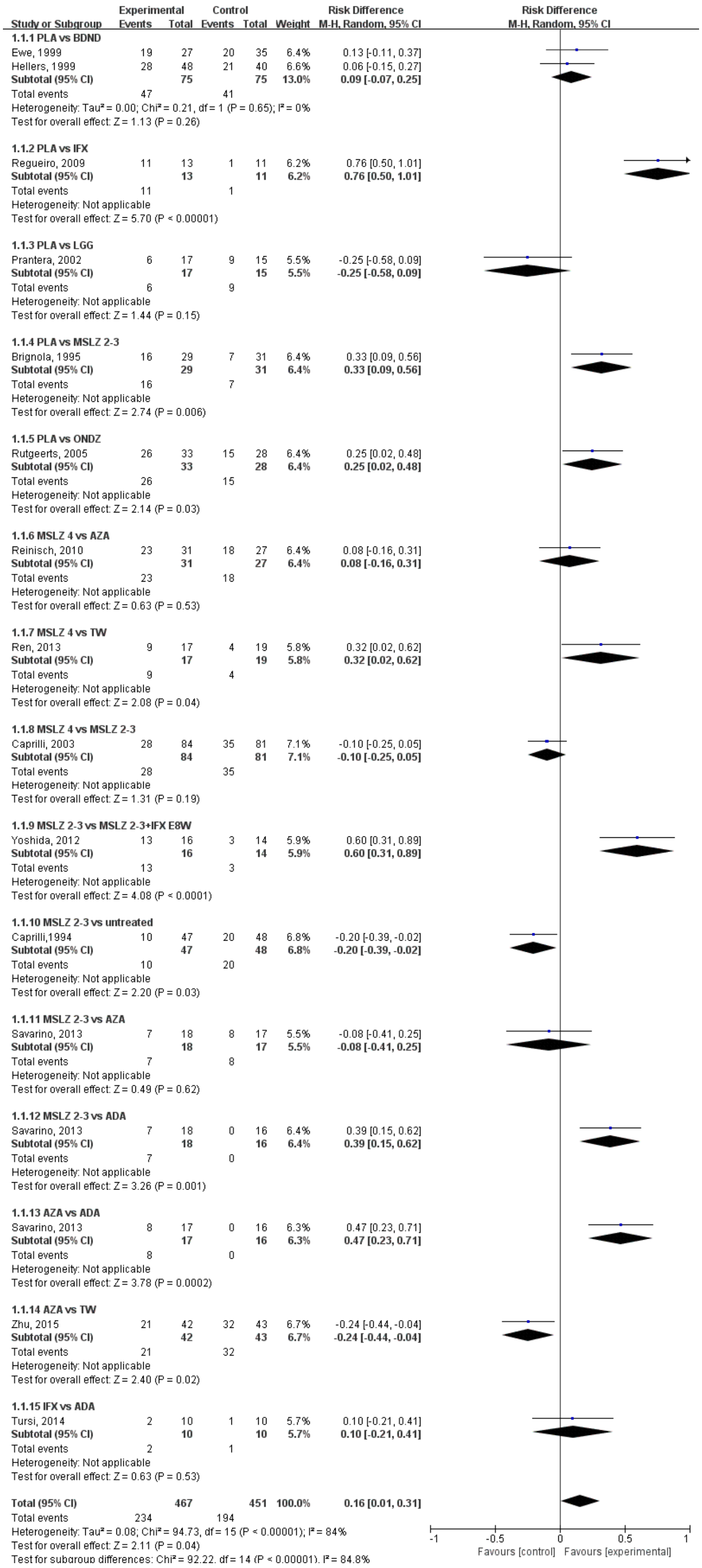

Figure 1. Forest plot

PLA, placebo; untreated, blank control group; MSLZ, mesalazine; BDND, budesonide; AZA, azathioprine; IFX, infliximab; ADA, adalimumab; TW, tripterygium wilfordii; LGG, lactobacillus GG.

2. Supplementary Methods

Table 4. Search strategy

Search	Add to builder	Query	Items found	Time
#4	Add	Search **(((((((intestinal resection) OR post-operative) OR recurrence) OR relapse) OR recur) AND ((Clinical Study[ptyp] OR Clinical Trial[ptyp] OR Clinical Trial, Phase I[ptyp] OR Clinical Trial, Phase II[ptyp] OR Clinical Trial, Phase III[ptyp] OR Clinical Trial, Phase IV[ptyp] OR Meta-Analysis[ptyp] OR Randomized Controlled Trial[ptyp])))) AND ((((((((((((((((Crohn Disease) OR Crohn's Enteritis) OR Regional Enteritis) OR Regional Ileitis) OR Regional Ileitides) OR Crohn's Disease) OR Crohns Disease) OR Inflammatory Bowel Disease 1) OR Enteritis, Granulomatous) OR Granulomatous Enteritis) OR Enteritis, Regional) OR Ileocolitis) OR Colitis, Granulomatous) OR Granulomatous Colitis) OR Ileitis, Terminal) OR Terminal Ileitis) OR Ileitis, Regional) AND ((Clinical Study[ptyp] OR Clinical Trial[ptyp] OR Clinical Trial, Phase I[ptyp] OR Clinical Trial, Phase II[ptyp] OR Clinical Trial, Phase III[ptyp] OR Clinical Trial, Phase IV[ptyp] OR Meta-Analysis[ptyp] OR Randomized Controlled Trial[ptyp])))** Filters: **Clinical Study; Clinical Trial; Clinical Trial, Phase I; Clinical Trial, Phase II; Clinical Trial, Phase III; Clinical Trial, Phase IV; Randomized Controlled Trial**	391	01:38:00
#3	Add	Search **(((((((intestinal resection) OR post-operative) OR recurrence) OR relapse) OR recur) AND ((Clinical Study[ptyp] OR Clinical Trial[ptyp] OR Clinical Trial, Phase I[ptyp] OR Clinical Trial, Phase II[ptyp] OR Clinical Trial, Phase III[ptyp] OR Clinical Trial, Phase IV[ptyp] OR Meta-Analysis[ptyp] OR Randomized Controlled Trial[ptyp])))) AND ((((((((((((((((Crohn Disease) OR Crohn's Enteritis) OR Regional Enteritis) OR Regional Ileitis) OR Regional Ileitides) OR Crohn's Disease) OR Crohns Disease) OR Inflammatory Bowel Disease 1) OR Enteritis, Granulomatous) OR Granulomatous Enteritis) OR Enteritis, Regional) OR Ileocolitis) OR Colitis, Granulomatous) OR Granulomatous Colitis) OR Ileitis, Terminal) OR Terminal Ileitis) OR Ileitis, Regional) AND ((Clinical Study[ptyp] OR Clinical Trial[ptyp] OR Clinical Trial, Phase I[ptyp] OR Clinical Trial, Phase II[ptyp] OR Clinical Trial, Phase III[ptyp] OR Clinical Trial, Phase IV[ptyp] OR Meta-Analysis[ptyp] OR Randomized Controlled Trial[ptyp])))** Filters: **Clinical Study; Clinical Trial; Clinical Trial, Phase I; Clinical Trial, Phase II; Clinical Trial, Phase III; Clinical Trial, Phase IV; Meta-Analysis; Randomized Controlled Trial**	469	01:35:58
#2	Add	Search **(((((((((((((((Crohn Disease) OR Crohn's Enteritis) OR Regional Enteritis) OR Regional Ileitis) OR Regional Ileitides) OR Crohn's Disease) OR Crohns Disease) OR Inflammatory Bowel Disease 1) OR Enteritis, Granulomatous) OR Granulomatous Enteritis) OR Enteritis, Regional) OR Ileocolitis) OR Colitis, Granulomatous) OR Granulomatous Colitis) OR Ileitis, Terminal) OR Terminal Ileitis) OR Ileitis, Regional** Filters: **Clinical Study; Clinical Trial; Clinical Trial, Phase I; Clinical Trial, Phase II; Clinical Trial, Phase III; Clinical Trial, Phase IV; Meta-Analysis; Randomized Controlled Trial**	2091	01:31:15
#1	Add	Search **((((intestinal resection) OR post-operative) OR recurrence) OR relapse) OR recur** Filters: **Clinical Study; Clinical Trial; Clinical Trial, Phase I; Clinical Trial, Phase II; Clinical Trial, Phase III; Clinical Trial, Phase IV; Meta-Analysis; Randomized Controlled Trial**	63992	01:25:01

Appendix:

Statistical code

```
# Binomial likelihood, logit link
# Fixed effects model
model{                              # *** PROGRAM STARTS
for(i in 1:ns){                     # LOOP THROUGH STUDIES
mu[i] ~ dnorm(0,.0001)              # vague priors for all trial baselines
for (k in 1:na[i]) {                # LOOP THROUGH ARMS
r[i,k] ~ dbin(p[i,k],n[i,k])        # binomial likelihood
```

```
logit(p[i,k]) <- mu[i] + d[t[i,k]] - d[t[i,1]]
                                        # model for linear predictor
rhat[i,k] <- p[i,k] * n[i,k]          # expected value of the numerators
dev[i,k] <- 2 * (r[i,k]*(log(r[i,k])-log(rhat[i,k]))
+(n[i,k]-r[i,k])*(log(n[i,k]-r[i,k])-log(n[i,k]-rhat[i,k])))
}                                       #Deviance contribution
resdev[i] <- sum(dev[i,1:na[i]])
                                        # summed residual deviance contribution for this trial
}
totresdev <- sum(resdev[])        #Total Residual Deviance
d[1]<-0                               # treatment effect is zero for reference treatment
for (k in 2:nt){ d[k] ~ dnorm(0,.0001) }
                                        # vague priors for treatment effects
}                                       # *** PROGRAM ENDS

# Binomial likelihood, logit link
# Random effects model for multi-arm trials
model{                                          # *** PROGRAM STARTS
for(i in 1:ns){                          # LOOP THROUGH STUDIES
w[i,1] <- 0                      # adjustment for multi-arm trials is zero for control arm
delta[i,1] <- 0                       # treatment effect is zero for control arm
mu[i] ~ dnorm(0,.0001)                   # vague priors for all trial baselines
for (k in 1:na[i]) {                       # LOOP THROUGH ARMS
r[i,k] ~ dbin(p[i,k],n[i,k])              # binomial likelihood
logit(p[i,k]) <- mu[i] + delta[i,k]               # model for linear predictor
rhat[i,k] <- p[i,k] * n[i,k]                     # expected value of the numerators
dev[i,k] <- 2 * (r[i,k] * (log(r[i,k])-log(rhat[i,k]))
+ (n[i,k]-r[i,k]) * (log(n[i,k]-r[i,k]) - log(n[i,k]-rhat[i,k])))    #Deviance contribution
}
resdev[i] <- sum(dev[i,1:na[i]])      # summed residual deviance contribution for this
trial
for (k in 2:na[i]) {                                    # LOOP THROUGH ARMS
delta[i,k] ~ dnorm(md[i,k],taud[i,k])                # trial-specific LOR distributions
md[i,k] <- d[t[i,k]] - d[t[i,1]] + sw[i,k]         # mean of LOR distributions (with
multi-arm trial correction)
taud[i,k] <- tau *2*(k-1)/k        # precision of LOR distributions (with multi-arm trial
correction)
w[i,k] <- (delta[i,k] - d[t[i,k]] + d[t[i,1]])    # adjustment for multi-arm RCTs
sw[i,k] <- sum(w[i,1:k-1])/(k-1)         # cumulative adjustment for multi-arm trials
}
}
totresdev <- sum(resdev[])                              #Total Residual Deviance
d[1] <- 0                                 # treatment effect is zero for reference treatment
for (k in 2:nt){ d[k] ~ dnorm(0,.0001) }                # vague priors for treatment effects
```

```
sd ~ dunif(0,5)                    # vague prior for between-trial SD.
ALTERNATIVES BELOW
tau <- pow(sd,-2)                              # between-trial precision =
(1/between-trial variance)
}                                        # *** PROGRAM ENDS
```

3. Included trials

[1] Hellers G, Cortot A, Jewell D, et al. Oral budesonide for prevention of postsurgical recurrence in Crohn's disease. The IOIBD Budesonide Study Group [J]. Gastroenterology, 1999, 116(2): 294-300.

[2] Ewe K, Böttger T, Buhr HJ, et al. Low-dose budesonide treatment for prevention of postoperative recurrence of Crohn's disease: a multicentre randomized placebo-controlled trial. German Budesonide Study Group [J]. European journal of gastroenterology & hepatology, 1999, 11(3): 277-282.

[3] Brignola C, Cottone M, Pera A, et al. Mesalamine in the prevention of endoscopic recurrence after intestinal resection for Crohn's disease. Italian Cooperative Study Group [J]. Gastroenterology, 1995, 108(2): 345-349.

[4] Regueiro M, Schraut W, Baidoo L, et al. Infliximab prevents Crohn's disease recurrence after ileal resection [J]. Gastroenterology, 2009, 136(2): 441-450.e441; quiz 716.

[5] Prantera C, Scribano ML, Falasco G, et al. Ineffectiveness of probiotics in preventing recurrence after curative resection for Crohn's disease: a randomised controlled trial with Lactobacillus GG [J]. Gut, 2002, 51(3): 405-409.

[6] Rutgeerts P, Assche G, Vermeire S, et al. Ornidazole for prophylaxis of postoperative Crohn's disease recurrence: a randomized, double-blind, placebo-controlled trial [J]. Gastroenterology, 2005, 128(4): 856-861.

[7] Caprilli R, Cottone M, Tonelli F, et al. Two mesalazine regimens in the prevention of the post-operative recurrence of Crohn's disease: a pragmatic, double-blind, randomized controlled trial [J]. Alimentary pharmacology & therapeutics, 2003, 17(4): 517-523.

[8] Reinisch W, Angelberger S, Petritsch W, et al. Azathioprine versus mesalazine for prevention of postoperative clinical recurrence in patients with Crohn's disease with endoscopic recurrence: efficacy and safety results of a randomised, double-blind, double-dummy, multicentre trial [J]. Gut, 2010, 59(6): 752-759.

[9] Ren J, Wu X, Liao N, et al. Prevention of postoperative recurrence of Crohn's disease: Tripterygium wilfordii polyglycoside versus mesalazine [J]. The Journal of international medical research, 2013, 41(1): 176-187.

[10] Yoshida K, Fukunaga K, Ikeuchi H, et al. Scheduled infliximab monotherapy to prevent recurrence of Crohn's disease following ileocolic or ileal resection: a 3-year prospective randomized open trial [J]. Inflammatory bowel diseases, 2012, 18(9): 1617-1623.

[11] Caprilli R, Andreoli A, Capurso L, et al. Oral mesalazine (5-aminosalicylic acid; Asacol) for the prevention of post-operative recurrence of Crohn's disease. Gruppo Italiano per lo Studio del Colon e del Retto (GISC) [J]. Alimentary pharmacology & therapeutics, 1994, 8(1): 35-43.

[12] Savarino E, Bodini G, Dulbecco P, et al. Adalimumab is more effective than azathioprine and mesalamine at preventing postoperative recurrence of Crohn's disease: A randomized controlled trial [J]. American Journal of Gastroenterology, 2013, 108(11): 1731-1742.

[13] Zhu W, Li Y, Gong J, et al. Tripterygium wilfordii Hook. f. versus azathioprine for prevention of postoperative recurrence in patients with Crohn's disease: a randomized clinical trial [J]. Digestive and liver disease : official journal of the Italian Society of Gastroenterology and the Italian Association for the Study of the Liver, 2015, 47(1): 14-19.

[14] Tursi A, Elisei W, Picchio M, et al. Comparison of the effectiveness of infliximab and adalimumab in preventing postoperative recurrence in patients with Crohn's disease: an open-label, pilot study [J]. Techniques in coloproctology, 2014, 18(11): 1041-1046.
